# Emotional food‐cue‐reactivity in anorexia nervosa and bulimia nervosa: An electroencephalography study

**DOI:** 10.1002/eat.24028

**Published:** 2023-08-10

**Authors:** Katharina Naomi Eichin, Claudio Georgii, Rebekka Schnepper, Ulrich Voderholzer, Jens Blechert

**Affiliations:** ^1^ Department of Psychology and Centre for Cognitive Neuroscience University of Salzburg Salzburg Austria; ^2^ Department of Psychology Johannes Kepler University Linz Austria; ^3^ Department of Psychosomatic Medicine University Hospital Basel Basel Switzerland; ^4^ Schoen Clinic Roseneck Prien am Chiemsee Germany

**Keywords:** anorexia nervosa, bulimia nervosa, electroencephalography, food‐cue‐reactivity, negative emotion

## Abstract

**Objective:**

Food‐cue‐reactivity entails neural and experiential responses to the sight and smell of attractive foods. Negative emotions can modulate such cue‐reactivity and this might be central to the balance between restrictive versus bulimic symptomatology in Anorexia Nervosa (AN) and Bulimia Nervosa (BN).

**Method:**

Pleasantness ratings and electrocortical responses to food images were measured in patients with AN (*n* = 35), BN (*n* = 32) and matched healthy controls (HC, *n* = 35) in a neutral state and after idiosyncratic negative emotion induction while electroencephalography (EEG) was recorded. The EEG data were analyzed using a mass testing approach.

**Results:**

Individuals with AN showed reduced pleasantness for foods compared to objects alongside elevated widespread occipito‐central food‐object discrimination between 170 and 535 ms, indicative of strong neural cue‐reactivity. Food‐object discrimination was further increased in the negative emotional condition between 690 and 1200 ms over centroparietal regions. Neither of these effects was seen in individuals with BN.

**Discussion:**

Emotion modulated food‐cue‐reactivity in AN might reflect a decreased appetitive response in negative mood. Such specific (emotion‐)regulatory strategies require more theoretical work and clinical attention. The absence of any marked effects in BN suggests that emotional cue‐reactivity might be less prominent in this group or quite specific to certain emotional contexts or food types.

**Public Significance:**

Negative affectivity is a risk factor for the development of eating disorders and individuals with eating disorders experience problems with emotion regulation. To better understand the effects of negative emotions, the present study investigated how they affected neural correlates of food perception in anorexia nervosa and bulimia nervosa.

## 1 INTRODUCTION

### 1.1 AN‐BN symptoms overlap/differences

Anorexia nervosa (AN) and bulimia nervosa (BN) share risk factors (Culbert et al., 2015) as well as core symptoms. These include attempts to restrict food intake and the high influence that eating, weight and body shape have on self‐worth (Duarte et al., 2016) and age of onset (Hudson et al., 2007). Furthermore, transitions between the two diagnoses (Schaumberg et al., 2019) as well as the “intermediate” binge‐purge subtype of AN, where both symptoms of AN and BN are present (American Psychiatric Association, 2013), point toward an intricate linkage between the two disorders. Despite these commonalities, AN and BN are not regularly contrasted in the context of food‐cue‐reactivity and mixed samples dominate (see Wolz et al., 2017).

### 1.2 Food‐cue‐reactivity

Food‐cue‐reactivity refers to psychophysiological and neural reactions to the sight and smell of foods and has become an umbrella term for processes that precede actual eating (Jansen, 1998). It has been explained through classical conditioning (Blechert et al., 2016; Jansen, 1998; Lender et al., 2020) and has been shown to predict food intake (Boswell & Kober, 2016; Nederkoorn & Jansen, 2002), overeating and binge‐eating (Jansen, 1998). Food‐cue‐reactivity is altered in individuals with AN, who give lower liking and wanting ratings for (high‐energy) foods, choose them less often in food‐decision tasks (Georgii et al., 2022; Lloyd & Steinglass, 2018) and, unsurprisingly, eat less in laboratory taste tests (Foerde et al., 2015). In behavioral tasks, they show neither an attentional bias (looking at foods longer than at objects; Giel et al., 2011) nor an approach bias to foods (Paslakis et al., 2016). They furthermore show stronger frowning responses of the corrugator muscle when looking at high‐energy foods, suggesting a negative emotional reaction (Schnepper et al., 2021). Thus, there is some indication for reduced cue‐reactivity on a behavioral level, although the picture is complicated (see the evidence on attentional biases in neural studies below). This might partially be a consequence of altered reward‐processing in AN, leading to the experience of food avoidance as rewarding (Keating et al., 2012). The picture is even less clear in BN: they rate foods similar to healthy controls and studies investigating their food choice behavior are inconsistent (Georgii et al., 2022; Gianini et al., 2019). However, emotional food‐cue‐reactivity studies point to a more appetitive response (higher desire to eat ratings, relaxed corrugator response; Schnepper et al., 2021). This would be consistent with the prominent observation of emotion related binge‐eating (Reichenberger et al., 2020) and reward learning in BN (i.e., the expectation that eating will alleviate negative emotions; Schaefer & Steinglass, 2021). Interestingly, while individuals with AN show hyperactivation in brain areas involved in top‐down control and hypoactivation in areas related to reward, individuals with BN show the opposite (Bronleigh et al., 2022) pointing toward disorder‐specific neural signatures.

### 1.3 Food‐cue‐reactivity in the electroencephalogram (EEG)

Another method to investigate food‐cue‐reactivity is EEG, affording high temporal resolution of neural processes. Most EEG studies compute event‐related potentials (ERPs), coarsely categorized into early and late components (Schupp et al., 2006). Early components are thought to be related to bottom‐up processes like attention (Luck & Kappenman, 2011) or biological significance/valence detection (Olofsson et al., 2008), while late components are thought to be related to stimulus appraisal (Hajcak & Foti, 2020), motivational attention and emotional reactivity (Hajcak et al., 2010). Individuals with AN show differences in an early time window (240–380 ms) in response to low‐energy foods, interpreted as increased attention, as well as in a later time window (400–700; Novosel et al., 2014). One study using magnetoencephalography (MEG) found increased activity peaking as early as 150 ms (Godier et al., 2016), in line with an early attentional bias to food. Therefore, in contrast to behavioral findings, neural findings point toward an attentional bias to food cues in AN. Early attentional processing, as visible in the EEG, might enable controlling later motivational processing and the translation into behavior (Blechert et al., 2011).

In BN, the literature on ERPs of food picture viewing is scarce. Women with bulimic symptomatology show an increased late positive potential (LPP; 500–800 ms) for binge foods, indicative of elevated emotional significance (Delgado‐Rodríguez et al., 2019). The only ERP‐study comparing AN and BN found both groups to differ from the control group in an early time window (220–310 ms), which was interpreted as facilitated processing of foods (Blechert et al., 2011). Participants with binge‐eating (the sample also included individuals with binge‐eating disorder (BED)) show a stronger early (180–350 ms) amplitude difference for pictures of chocolate as compared to the control group, which might be related to inhibitory control and response conflict (Wolz et al., 2017). Individuals with BED who share the binge symptomatology with individuals with BN show larger amplitudes for high‐energy foods in late time windows (500–800 ms, 1000–6000 ms; Svaldi et al., 2010), something the authors attributed to the allocation of attention and motivation. Taken together, ERPs show altered food‐cue‐reactivity in both AN and BN, for early and late time windows. However, the number of studies is limited, the time windows of interest vary considerably and direct comparison of the two groups is mostly lacking.

### 1.4 Negative emotion

Most of the findings discussed above refer to cue‐reactivity in response to food images under “neutral” conditions. But what happens when emotions come into play? Research suggests that eating might function as a means of downregulating negative emotions (Macht & Simons, 2011). Negative affect is a common characteristic in eating disorders (Hilbert et al., 2014) and both AN and BN have difficulties with emotion regulation (Svaldi et al., 2012). In line with this idea, the frequency of binge‐purge behavior is elevated on days with increased negative effect (Haedt‐Matt & Keel, 2011; Reichenberger et al., 2020) and negative emotions are related to food restriction on the following day (Engel et al., 2013). Following a sadness manipulation in the laboratory, individuals with AN and BN that used rumination, a dysfunctional emotion regulation strategy, showed an increase in eating‐related symptoms (Naumann et al., 2015; Naumann & Svaldi, 2021). Individuals with AN responded with a desire to abstain from eating, while individuals with BN responded with a desire to binge (Naumann et al., 2015). The only study investigating the effect of emotion on ERPs in response to food found individuals with BN to show a nonspecific effect of emotion on visual processing (i.e., enhanced activity to both foods and control images) in an early window (350–400 ms) and an emotion‐specific effect for high‐calorie foods in a late (600–1000 ms) time window (Lutz et al., 2021). Individuals high on emotional eating, which is discussed as a risk factor for disordered eating (Sultson & Akkermann, 2019), show a late effect of emotion (300–600 ms) for food images (Blechert et al., 2014). Other ERP studies investigating the effect of emotions in individuals with eating disorders found reduced neural processing of emotional faces in AN (Pollatos et al., 2008; Sfärlea et al., 2016). In contrast, emotional face processing was increased in BN (Kühnpast et al., 2012). To sum up, although there is some indication that negative emotions might affect food processing in AN and BN, there is limited experimental evidence for effects of emotion on food‐cue‐reactivity.

### 1.5 Present study and hypotheses

The current study investigated cue‐reactivity to food in AN, BN and healthy controls (HC) and the role of negative emotion. Participants viewed pictures of foods and objects in a neutral and an emotionally negative condition while EEG and pleasantness ratings were recorded. In parallel to the rating data published in Schnepper et al. (2021), we expected reduced pleasantness of food in AN and increased pleasantness of food in BN. Neurally, we expected increased electrocortical discrimination of foods and objects (i.e., difference in the influence of image type on the EEG‐amplitude) in both AN and BN in an early time window (100–300 ms; g*eneral food‐cue‐reactivity hypothesis*) as previous studies found an increase in components related to attentional processing (Blechert et al., 2011; Novosel et al., 2014). Additionally, we expected an effect of negative emotion on food‐cue‐reactivity in individuals with BN as expressed in increased food pleasantness ratings and food‐object discrimination in the emotion condition. As previous research found an increased desire to abstain from eating for negative emotions and dysfunctional emotion regulation (Naumann et al., 2015), and decreased processing of emotional faces (Sfärlea et al., 2016) in AN, we expected the opposite in this group: *de*creased food pleasantness ratings and food‐object discrimination in the emotion condition (*emotional food‐cue‐reactivity hypothesis*). We anticipated such emotion effects in a later time window (>300 ms), where emotional and motivational reactivity are reflected (Hajcak et al., 2010).

## 2 METHODS

### 2.1 Participants

*N* = 87 women (sex assigned at birth) without eating disorders (*n* = 5 underweight, *n* = 64 normal weight, *n* = 18 overweight), *n* = 42 women with AN and *n* = 40 with BN participated in the study which was part of a larger project (see supplement). Participants without eating disorders took part in the study at the University of Salzburg, Austria; patients with AN or BN took part in a major German treatment center for eating disorders (Schoen Klinik, Prien am Chiemsee, Germany). The experimental equipment and set‐up were identical at both sites. Exclusion criteria for the control group were: past and present eating disorders, vegetarianism or veganism, relevant food or skin allergies, neurological or psychotic disorders, pregnancy, diabetes and current substance abuse. Participants were also excluded if they had less than 70% valid epochs in the EEG. For details on data exclusion see supplement. After exclusions the sample consisted of *n* = 71 (*n* = 4 underweight, *n* = 64 normal weight, *n* = 13 overweight) HC, *n* = 35 participants with AN and *n* = 32 participants with BN. For the final sample, to have approximately equal group sizes, a random sample of *n* = 35 participants without eating disorders was drawn (*n* = 1 underweight, *n* = 27 normal weight, n = 7 overweight; for a sample characterization see Table 1). Groups were age‐ and education‐matched. *N* = 79 participants were German, *n* = 21 Austrian, *n* = 1 Italian and *n* = 1 of unknown nationality. The study was approved by the University of Salzburg Ethics committee and the University of Munich medical review board.

**TABLE 1:** Sample characterization.

	*M* (*SD*)			
	HC (*n* = 35)	AN (*n* = 35)	BN (*n* = 32)	ANOVA
Age (in years)	23 (4.65)	22.66 (5.22)	24.81 (8.07)	*p* = .31
BMI	22.51 (2.79)	15.53 (1.79)	22.81 (3.52)	*p* < .001; HC = BN > AN
Education (in years)	15.04 (2.97)	14.54 (2.73)	13.86 (2.75)	*p* = .27
Hunger	4 (1.71)	1.94 (1.28)	2.69 (1.75)	*p* < .001; HC > AN = BN
Eating Pathology (EDE‐Q)	1.73 (1.22)	3.29 (1.45)	4.2 (1.34)	*p* < .001; HC < AN < BN
Depression	9.77 (5.9)	23.03 (8.81)	20.59 (12.59)	*p* < .001; HC < AN = BN
Anxiety	41.09 (11.1)	56.54 (9.46)	55.66 (12.6)	*p* < .001; HC < AN = BN
Negative affect neutral condition	12.17 (2.48)	14.2 (4.3)	17.41 (9.14)	Neutral vs. negative: *p* < .001
Negative affect negative condition	16.49 (5.77)	20.4 (7.18)	24.31 (11.06)	

*Note*: Mean (*M*) and standard deviation (*SD*) of sample characteristics. The ANOVA column indicates which groups differ significantly. Negative affect was measured using the positive and negative affect schedule (PANAS; Krohne et al., 2016). Depressive symptoms were measured using the ADS‐K (Hautzinger & Bailer, 1993). Anxiety was measured using the STAIT (Laux et al., 1981). Eating pathology was measured using the Eating Disorder Examination‐Questionnaire (EDE‐Q; Hilbert et al., 2007).

### 2.2 Procedure

#### 2.2.1 Structured clinical interview

All participants underwent two clinical interviews (Eating Disorder Examination and the Structured Clinical Interview for DSM‐IV; Hilbert & Tuschen‐Caffier, 2016; Wittchen et al., 1997) to allow diagnosis in line with DSM‐5 (American Psychiatric Association, 2013).

#### 2.2.2 Pre‐laboratory procedure

Laboratory testing was scheduled at 3 pm for all participants. To additionally limit hunger differences, participants without eating disorder were asked to consume one of five standardized lunches (~550 kcal) at noon and not to eat between lunch and the testing. The groups with AN and BN followed their regular eating schedule in the treatment center.

#### 2.2.3 Idiosyncratic interview for induction of negative emotion

Idiosyncratic emotion induction was similar to previous studies (Blechert et al., 2014; Hilbert et al., 2011): participants were asked to report recent events in which they experienced negative emotions. They were asked to rate how negative those events were, how much distress they felt recalling them and how well they remembered them. The event with the highest ratings was explored in further detail. The experimenter then created a script of eight sentences which were later presented during the task to induce negative emotion. For a neutral control condition, participants chose between two prescripted situations that described going to university, work or school or brushing teeth. A manipulation check using the negative subscale of the PANAS (Krohne et al., 2016) showed successful negative emotion induction, with comparable strength in all groups (see Table 1).

#### 2.2.4 Emotional cue‐reactivity task

**FIGURE 1:**
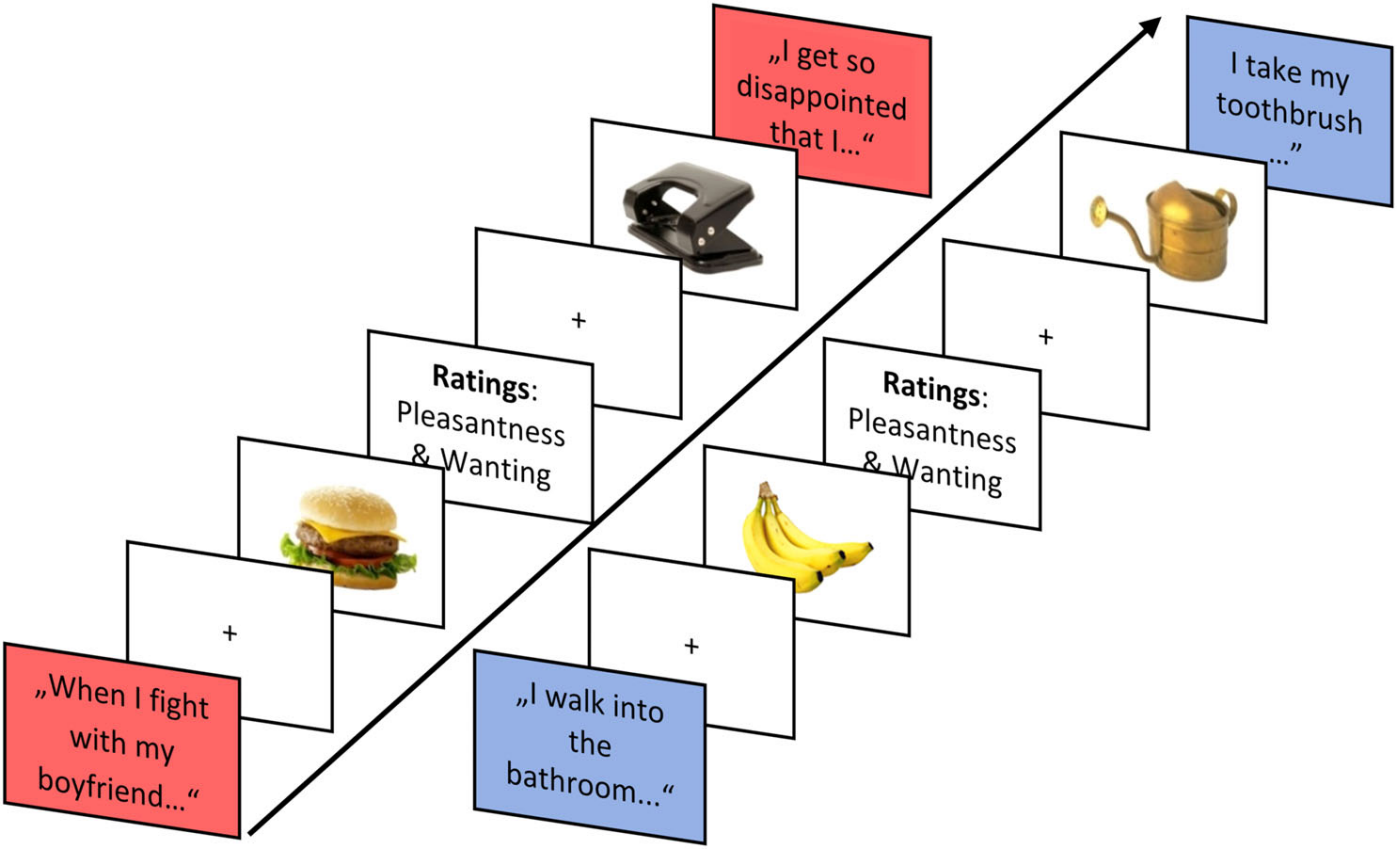
Emotional food‐cue‐reactivity task. Visualization of the emotional picture viewing task. Depending on the condition, emotional or neutral sentences were interleaved with pictures of foods and objects. Additionally, images were rated on pleasantness and/or desire to eat once per block.

The emotional picture viewing task (see Figure 1) consisted of an emotionally negative and a neutral block. Block order was randomized between participants. At the beginning of each block the pre‐created scripts were read to the participants and they were asked to reimagine the situation. Twenty‐six images of foods and 26 images of objects were presented twice per block (in total 208 trials). Images were taken from a validated database (food‐pics database Blechert et al., 2019) and matched based on image characteristics (see supplement). Image presentation was interleaved with presentation of the sentences from the scripts to keep the situations in memory. Images were rated for pleasantness (from 0 “very unpleasant” to 100 “very pleasant”) and desire to eat (foods only) on a visual analogue scale (0–100) once per block. Each block was followed by PANAS ratings (Krohne et al., 2016). EEG was recorded during picture presentation. The task took approximately 40–50 min to complete.

### 2.3 EEG recording and data analysis

EEG was recorded using a 64‐channel amplifier and a 63‐channel equidistant cap (sintered Ag/AgCl electrodes; TMSi, Twente Medical Systems International, EJ Oldenzaal, Netherlands) with a ground electrode attached at the left wrist. The signal was sampled at 512 Hz; impedances were kept below 10 kΩ. Preprocessing was done in EEGLab (Delorme & Makeig, 2004). Data were down‐sampled to 256 Hz and filtered using a low‐pass filter at 70 Hz, a high‐pass‐filter at 0.1 Hz and a notch‐filter at 45–55 Hz. Noisy channels were removed after visual inspection. Data was re‐referenced to average reference. Eye‐blink and ‐movement artifacts were removed in an independent component analysis using AMICA (Palmer et al., 2011). The signal was epoched from 1500 ms pre‐stimulus to 2400 ms post‐stimulus. Epochs with large artifacts were excluded upon visual inspection. Removed channels were interpolated.

#### 2.3.1 Rating data analysis

Pleasantness ratings given during picture viewing were analyzed with a repeated measures ANOVA using image type (foods vs. objects), emotion (neutral vs. negative) and group (HC, vs. AN, vs. BN) as predictors. The analysis was done using the rstatix package (Kassambara, 2021).

#### 2.3.2 EEG data analysis

A mass testing analysis was performed in the LIMO EEG toolbox (Pernet et al., 2011) for Matlab R2021a (The MathWorks Inc., Natick, MA) using a hierarchical linear modeling approach. For the rationale of using a mass testing approach see supplement. All electrodes were investigated at all sampling points. At the first level (subjects), a general linear model with four regressors (equivalent to the four combinations of emotion and image type: neutral foods, neutral objects, negative foods, negative objects) was set up to obtain beta values for the influence of the conditions on the ERP. Variables were imported from EEGLAB and automatically extracted and processed (Bellec et al., 2012) for all participants on a single trial level for the time period −50 to 1200 ms. At the second level (group) a repeated measures ANOVA for group (HC, AN, BN) × image type (foods vs. objects) × emotion (negative vs. neutral) was computed. Results were corrected for multiple comparisons using spatio‐temporal clustering with a cluster‐forming threshold of *p* = .05 and bootstrapping (*n* = 1000). This hierarchical approach is similar to the one commonly used in fMRI analysis, for example, in SPM (Pernet et al., 2015). Thus, the *general food‐cue‐reactivity analysis* was done on spatio‐temporal clusters resulting from image type × group interactions, whereas the *emotional food‐cue‐reactivity analysis* was done on clusters resulting from emotion × image type × group interactions.

As the toolbox does not allow for between‐group post‐hoc tests, results (beta‐values) from the first‐level analysis were exported to R (R Core Team, 2021) and analyzed with the packages brms (Bürkner, 2017) and emmeans (Lenth, 2021). For each significant cluster, beta‐values for all electrodes and timepoints involved in that cluster were extracted. Beta‐values indicate the magnitude of influence a variable has on the outcome, such that negative betas indicate lower and positive betas indicate higher amplitudes. Robust linear models using a student‐*t* distribution were followed up with Tukey‐corrected contrasts. Weakly informative brms default priors were used. Bayesian analyses give *β*‐values and credible intervals (CI) instead of classical *p*‐values. CIs indicate the range in which the estimate lies with a probability of 95%. In addition, *p*‐directions (*pd*s) were calculated using the insight package (Lüdecke et al., 2019). *Pd*s take on values between 50% and 100% and represent the closest correspondence to a *p*‐value in the Bayesian framework. They indicate the probability that the effect lies in the direction that the estimate indicates (Makowski et al., 2019). All models converged with Rhat < 1.01, ESS > 1500 and no divergent transitions.

## 3 RESULTS

### 3.1 Pleasantness ratings

The image type × emotion × group ANOVA for pleasantness ratings yielded significant two‐way‐interactions for image type × group (*F*(2,99) = 13.13, *p* < .001, *η* ^2^ = .21) and emotion × group (*F*(2,99) = 6.57, *p* = .002, *η* ^2^ = .12). As expected under the *general food‐cue‐reactivity hypothesis*, Bonferroni‐corrected post‐hoc tests showed that foods were rated as more pleasant than objects in BN (*p* < .001) and HC (*p* < .001) but not in AN (*p* = .9, image type × group interaction). Negative emotion decreased pleasantness ratings of both foods and objects in AN (*p* = .04) and HC (*p* = .01) but not in BN (*p* = .99, emotion × group).

**FIGURE 2:**
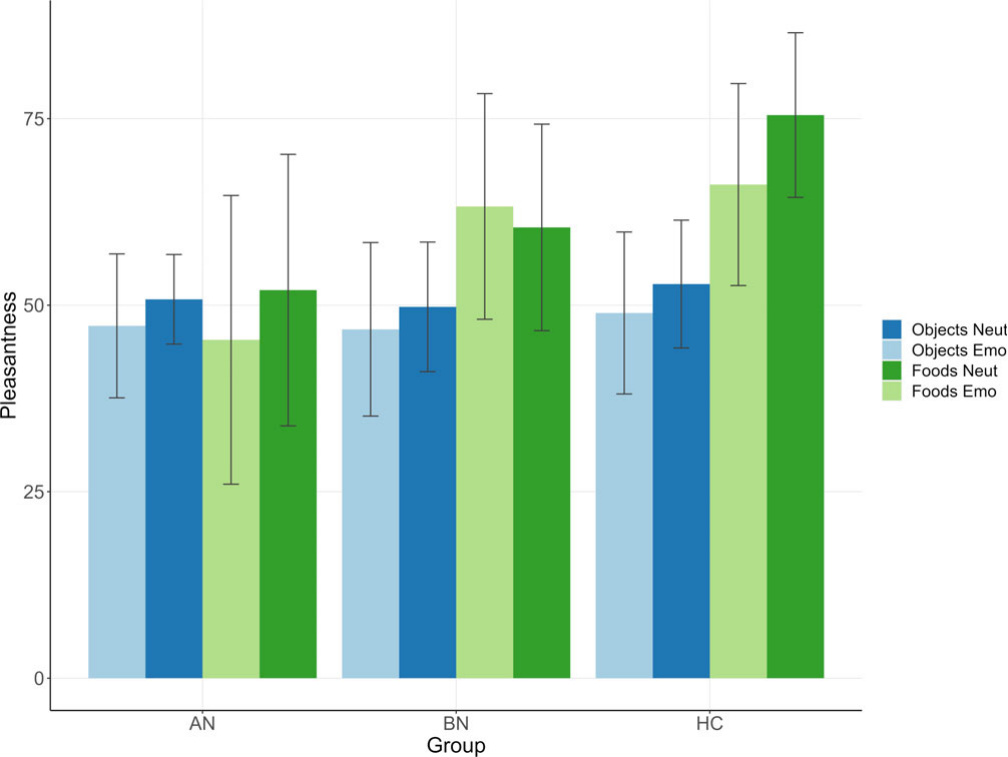
Pleasantness ratings for foods and objects in the neutral and negative condition. Pleasantness ratings for foods and objects on a visual analogue scale from 0 to 100 in the neutral and negative condition. HC = healthy controls (*n* = 35); AN = anorexia nervosa (*n* = 35); BN = bulimia nervosa (*n* = 35).

In contrast to what was expected in the *emotional food‐cue‐reactivity hypothesis*, post‐hoc tests for the significant 3‐way interaction (image type × emotion × group, *F*(2, 99) = 6, *p* = .003, *η* ^2^ = .11) revealed a significant effect of emotion only in HC (*p* = .002), with foods being rated as less pleasant in negative emotional state, but not in AN or BN. See Figure 2, Tables S1 and S2.

### 3.2 EEG results

#### 3.2.1 General food‐cue hypothesis

**FIGURE 3:**
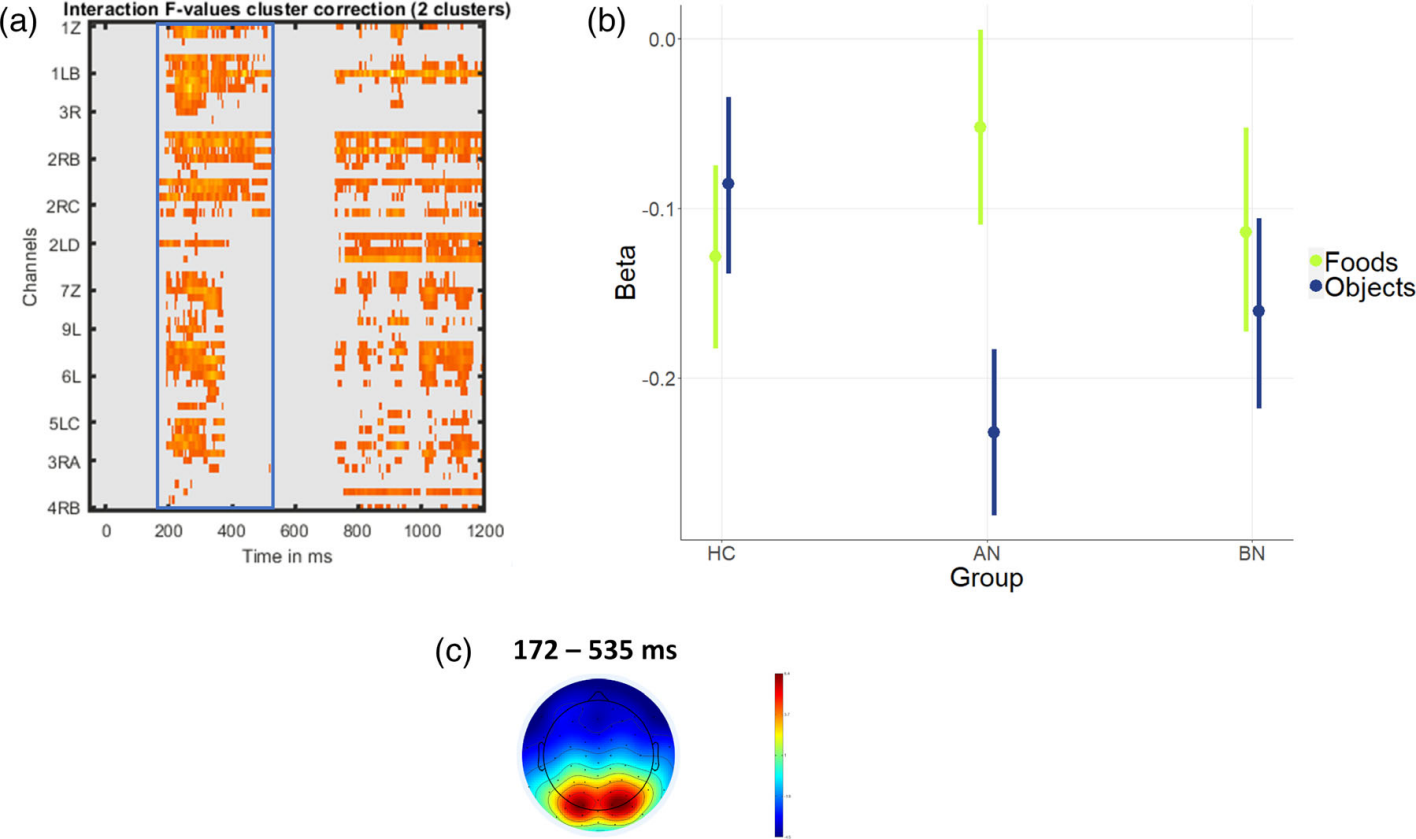
General food‐cue‐reactivity hypothesis (early cluster). (a) Results of the EEG mass‐testing analysis for the image type × group interaction. The blue frame highlights the early cluster analyzed for the general food‐cue hypothesis. (b) Results of the linear model for the early cluster (blue frame). Beta values indicate the magnitude of influence of conditions on EEG amplitude. (c) Topoplot for the the time window of the early cluster. HC = healthy controls (*n* = 35); AN = anorexia nervosa (*n* = 35); BN = bulimia nervosa (*n* = 32).

EEG mass testing of the group × image type interaction yielded two large significant clusters. The ‘early’ cluster encompassed the time points from 172 to 535 ms (see Figure 3, blue box) peaking over occipital electrodes. Tukey‐corrected post‐hoc contrasts for foods vs. objects showed the expected difference in the group with AN (*Δ* = 0.18, *CI* = [0.11; 0.26], *pd* = 100%). Amplitudes were more negative for objects compared to foods, suggesting an increased food‐object differentiation in early ERP components. In contrast to expectations, in BN (*Δ* = 0.05, *CI* = [−0.03; 0.13], *pd* = 87.55%; Tables S3 and S4) food‐object differences were small and only reached a *pd* of below 90%, indicating a lower effect probability (below 95%). The same was true in HC (*Δ* = −0.04, *CI* = [−0.12; 0.03], *pd* = 86.23%). A significant late cluster (727–1199 ms) was considered in the context of the three‐way interaction (group × image type × emotion) in the next step.

#### 3.2.2 Emotional food‐cue hypothesis

**FIGURE 4:**
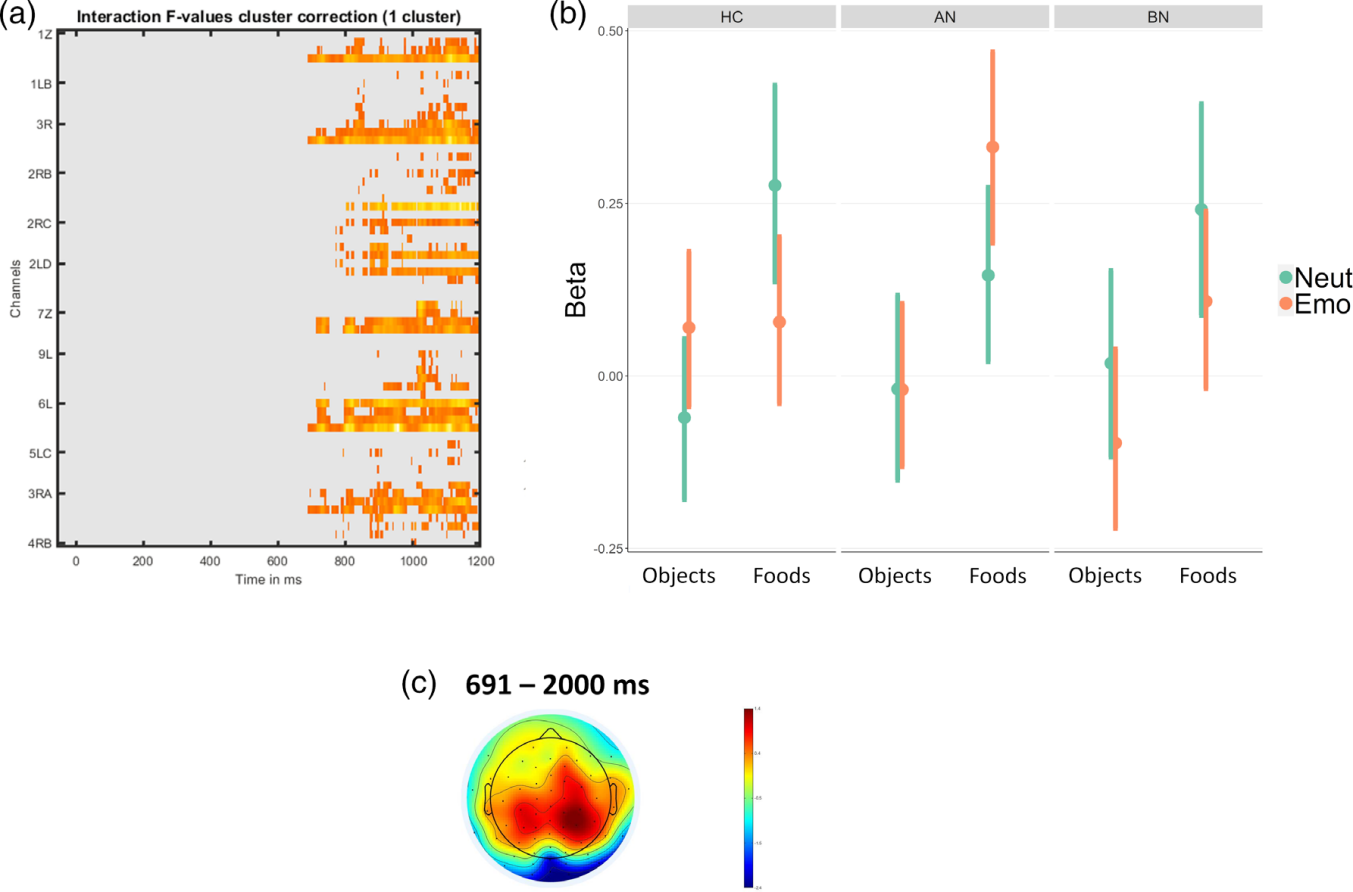
Emotional food‐cue‐reactivity hypothesis. (a) Results of the EEG mass‐testing analysis for the image type × emotion × group interaction. (b) Results of the linear model for the extracted cluster. Beta values indicate the magnitude of influence of conditions on EEG amplitude. (c) shows the topoplot for the cluster time window. HC = healthy controls (*n* = 35); AN = anorexia nervosa (*n* = 35); BN = bulimia nervosa (*n* = 32).

Mass testing for the group × image type × emotion interaction revealed one late significant centro‐parietal cluster spanning from 691 to 1199 ms, therefore in the LPP time range (Figure 4, Table S5). Tukey‐corrected post‐hoc contrasts were calculated for the *neutral*‐ *vs. negative* condition for each group and image type (foods and objects) separately. Other than expected, for individuals with BN, emotion did not only affect foods *(Δ* = 0.13, *CI* = [−0.07; 0.33], *pd* = 90.48%) but also objects (*Δ* = 0.12, *CI* = [−0.07; 0.29], *pd* = 88.48%). Amplitudes were more positive in the neutral condition compared to the negative condition, indicating a generally decreased ERP in the negative condition. Both effects were small and of lower probability (*pd* below 95%). In AN, an effect of emotion was seen for foods, with higher amplitudes in the negative compared to the neutral condition, suggesting an effect of negative emotion on food processing (*Δ* = −0.19, *CI* = [−0.37; 0.00], *pd* = 97.3%). No effect of emotion was seen for objects (*Δ* = −0.00, *CI* = [−0.18; 0.18], *pd* = 50.25%). In the HC group there was an effect of emotion on both objects and foods but in opposite directions: for objects amplitudes were increased in the negative condition while for foods amplitudes were decreased, suggesting lower food‐object discrimination in the negative condition (objects: *Δ* = −0.13, *CI* = [−0.29; 0.03] *pd* = 94.73%; foods: *Δ* = 0.2, *CI* = [0.01; 0.38], *pd* = 98.05%). See Table S6.

## 4 DISCUSSION

The present EEG study was the first to contrast individuals with AN and individuals with BN on emotional food‐cue‐reactivity. Pleasantness ratings confirmed our *general food‐cue‐reactivity hypothesis* in that AN showed reduced food pleasantness ratings. Similarly, the expected early food‐object differentiation was seen also neurally in AN. Yet, unexpectedly, in BN and HC less evidence for food‐object differentiation was seen. Further, data did not support our *emotional food‐cue‐reactivity hypothesis* in *BN*: the expected increase in food pleasantness alongside neural food‐object discrimination in the negative condition was not seen in BN. Instead, individuals with AN showed an increased neural food‐object‐discrimination in a late time window in negative emotional state.

### 4.1 General food‐cue‐reactivity

The finding of robust neural food‐cue‐reactivity in individuals with AN is in line with previous findings of altered ERPs for foods in early time windows (Blechert et al., 2011; Godier et al., 2016; Novosel et al., 2014). The cluster is relatively large in temporal as well as in spatial terms. As such, several processes are likely involved in its generation. Early ERP signal between 150 and 200 ms is typically linked to attention (Luck, 2014), as is the time between 200 and 300 ms which is also related to affective processing of visual stimuli. The time window between 300 and 500 ms is seen as associated with the motivational significance of stimuli (Hajcak et al., 2011). It is thus likely that the observed beta (i.e., amplitude) difference between foods and objects in individuals with AN is driven by both attentional and emotional‐motivational aspects of food perception. One could speculate that this neural food‐object discrimination represents a “struggle” of individuals with AN to control the potential threat from appetitive foods, for example, through devaluation, which might be reflected in reduced pleasantness ratings.

The absence of a clear BN‐specific food‐cue‐reactivity in our data is consistent with Delgado‐Rodríguez et al. (2019), who used images of individualized “binge‐foods” and found no early difference relative to HC. This suggests that foods that trigger cue‐reactivity in BN might be quite specific, whereas individuals with AN show a quite broad reactivity. Blechert et al. (2011) on the other hand documented cue‐reactivity in BN; however only in very early neural components that emerge in rapid serial presentation designs and that are thus not necessarily comparable with our paradigm.

### 4.2 Emotion‐modulated food‐cue‐reactivity

We expected to see emotional food‐cue‐reactivity in individuals with BN, visible in higher ratings for foods in the negative condition and in a higher food‐object difference in a late time window in the EEG. Neither of these two hypotheses was supported. This is surprising, given the centrality of emotional eating in the symptomatology of BN and the results of emotional eating questionnaires on this matter (Meule et al., 2021; Reichenberger et al., 2021). The lack of a clear effect might be due to the fact that our food stimuli included high‐ as well as low‐calorie foods, which we combined in the analysis to maximize statistical power and signal smoothness. It is conceivable that individuals with BN show increased cue‐reactivity only for high‐energy foods, as these imply a higher vulnerability for triggering binge‐eating. Preliminary analyses indeed show small effects for high‐calorie foods on a descriptive level (see Tables S7 and S8, Figures S1 and S2). Lutz et al. (2021) used the same task as the present study in a smaller BN sample (*n* = 21) and showed an emotional modulation of late cortical potentials to high‐calorie food‐cues. Yet, their findings emerged in quite circumscribed electrode locations, leaving some uncertainty about the robustness of the findings. Thus, follow‐up studies should increase the trial power for high calorie foods, for example, by splitting the emotion induction between two sessions. If anything, our finding of generally suppressed electrocortical reactivity to both foods and objects under negative emotions in BN might point to additional processes that divert attention from the foreground stimuli.

Unlike in BN, a food specific effect of negative emotion was seen in AN. Because late potentials in the ERP are known to be increased for biologically relevant stimuli (Hajcak et al., 2011) it would be plausible that for individuals with AN, who are significantly underweight, foods would have high significance. This coheres with findings that AN report to eat less when in negative mood (Meule et al., 2021; Reichenberger et al., 2021) and report a desire to abstain from eating when using dysfunctional emotion regulation strategies (Naumann et al., 2015). These results suggest that decreasing appetitive responses serve individuals with AN to reduce negative emotion, potentially through decreasing fear of weight gain (see Murray et al., 2018). The quite late emergence of emotion modulation might reflect this desire to restrain food intake, a phenomenon that deserves more theoretical and clinical attention.

### 4.3 Strengths, limitations and future directions

This study was the first EEG study to compare emotional food‐cue‐reactivity in individuals with AN and BN. Strengths include multiple controls, that is, neutral, nonemotional food‐cue‐reactivity assessment, inclusion of neutral objects, well matched groups and the inclusion of participants that fulfilled the entire diagnostic criteria in the clinical groups. The novel mass‐testing approach allowed for investigating effects of emotional food‐cue‐reactivity across the whole time and electrode spectrum, while controlling for false positive findings. While this is a strength because the problem of implicit multiple comparisons (Luck & Gaspelin, 2017) that comes with extracting data where effects are visible is avoided, it also makes comparisons with previous studies more difficult. The significant clusters in this study are larger, both in time and electrode‐space, than the spatio‐temporal regions of previous studies. Further, we did not compare ERP amplitude directions, as those depend on factors like EEG‐montage and reference. Instead, we compared beta‐values of the *influence* that different conditions had on the ERP for each person individually. Concerning the sample, to match the BMI range in the group with BN, no BMI‐criterion was applied in the control group. This might limit comparability with other studies including only individuals with normal weight. In addition, although samples were larger than in previous EEG studies in populations with eating disorders, sample sizes were still constrained by the availability of patients with AN and BN. Future studies should therefore replicate our findings based on a‐priori sample size calculations. Last, while the idiosyncratic emotion induction used in our study was successful (see Table 1) and ensured personal relevance it also allowed for variance in the type of emotions induced. Different negative emotions might vary in their effects on food‐cue‐reactivity which would be blurred in our results. Therefore, future studies might focus on comparing the effects of more specific negative emotions on cue‐reactivity as well as on individualized predictors of eating‐disordered behaviors (see Arend et al., 2023).

To conclude, our findings emphasize the relevance of negative emotions in food processing in AN. Therefore, supporting individuals with AN in developing their emotion regulation skills might help improve eating pathology (Rowsell et al., 2016). Theoretical and practical work should pay more attention to emotion‐related alterations in appetitive processing in this group and determine whether this pattern might be a mechanism that maintains the disorder or that is malleable through treatment. For BN, this study showed that symptoms of uncontrolled eating episodes as in binge eating do not seem to have a specific neural signature when considering high‐ and low‐calorie foods together. Future research is needed to determine the conditions in which negative emotions affect food‐cue processing along with the role of general emotional reactivity.

## AUTHOR CONTRIBUTIONS

**Katharina Naomi Eichin:** Data curation; formal analysis; methodology; visualization; writing – original draft; writing – review and editing. **Claudio Georgii:** Conceptualization; data curation; formal analysis; investigation; project administration. **Rebekka Schnepper:** Formal analysis; investigation; project administration; writing – review and editing. **Ulrich Voderholzer:** Resources. **Jens Blechert:** Conceptualization; funding acquisition; project administration; resources; supervision; writing – review and editing.

## FUNDING INFORMATION

This work was supported by the European Research Council (ERC) under the European Union's Horizon 2020 research and innovation program (ERC‐StG‐2014 639445 NewEat) and the Doctoral College “Imaging the Mind” (FWF; W1233‐B).

## CONFLICT OF INTEREST STATEMENT

The authors have no conflict of interest to declare.

### OPEN RESEARCH BADGES

This article has earned an Open Data badge for making publicly available the digitally‐shareable data necessary to reproduce the reported results. The data is available at https://doi.org/10.17605/OSF.IO/KC9G5.

## Supporting information


**Data S1.** Supporting Information

## Data Availability

Data is available at OSF: https://doi.org/10.17605/OSF.IO/KC9G5.
