# Supplementary material for: Emotional food‐cue‐reactivity in anorexia nervosa and bulimia nervosa: An electroencephalography study
Source: Int J Eat Disord. 2023 Aug 10;56(11):2096–106. doi: 10.1002/eat.24028 (PMC10946739; doi:10.1002/eat.24028)
Supplement: Supplementary file 1 — Data S1. Supporting Information [file EAT-56-2096-s001.docx]

**Supplement**

The data used for this manuscript is part of a larger project consisting of several tasks. As such, other papers with a partial sample overlap have been already published. Below is a list with papers from the project that have a partial overlap in data or sample. Three papers on emotional food-cue reactivity have been published: one paper reports EEG findings with a partial data overlap of healthy control participants (DOI: 10.3389/fnbeh.2020.00091), two papers on EMG results with a partial data overlap of rating data (DOI: 10.1002/erv.2849) and (DOI: 10.1002/eat.23683).

Three paper from an unrelated task concerning decision making have partial overlap in samples (DOI: 10.1007/s00426-019-01185-3; DOI: 10.1016/j.appet.2021.105745 ; 10.1016/j.appet.2021.105890).

Four papers have a partial sample overlap but concern unrelated tasks focusing on questionnaires on emotional eating and emotion regulation (DOI:10.1080/10640266.2019.1642036; DOI: 10.1017/S0029665120007004, DOI: 10.1002/eat.23477) and interoceptive sensitivity (DOI: 10.1002/erv.2676).

**Stimulus matching**

Stimuli were matched based on colour (red, green, blue), object size, intensity, SD, complexity, normalized complexity and median power (available from the foodpics database; Blechert et al., 2019).

**Data exclusions**

N = 8 participants ( 8 HC / 0 AN / BN ) were excluded because of technical problems during recording, n = 4 participants ( 4 / 0 / 0) were excluded because they reported eating disorder symptoms in the screening, n = 16 (3 / 7 / 6) participants were excluded because of few usable epochs, n = 1 (1 / 0 / 0) participant was excluded because of current substance abuse, n = 1 ( 0 / 0 / 1) because she was disgusted by food, n = 1 because of atypical bulimia, leaving n = 71 (n =4 underweight, n = 64 normal weight, n = 13 overweight) healthy controls, n = 35 participants with AN and n = 32 participants with BN.

**Mass testing approach**

As the few studies investigating food ERPs targeted a variety of different time windows and electrode positions, only one study contrasted AN and BN, and only one study in healthy participants investigated the influence of negative emotion, it was difficult to predict when and where effects were to be expected. For that reason, instead of setting a priori hypotheses on electrodes and time windows, which might overlook effects (Groppe et al., 2011) or deciding where to extract data by looking at the plots, which raises the problem of implicit multiple comparisons (Luck & Gaspelin, 2017), we employed a data driven mass testing approach. Mass testing examines the effects of interest at all electrodes and all relevant time points while correcting for multiple testing (Groppe et al., 2011).

**sTable 1**

*Pleasantness ratings*

| Predictor | *df_Num_* | *df_Den_* | *SS_Num_* | *SS_Den_* | *F* | *p* | *η^2^* |  |
| --- | --- | --- | --- | --- | --- | --- | --- | --- |
| (Intercept) | 1 | 99 | 1228365.19 | 25999.00 | 4677.42 | .000 |  |  |
| Group | 2 | 99 | 10104.24 | 25999.00 | 19.24 | .000 | .28 |  |
| Emo | 1 | 99 | 1579.15 | 5766.18 | 27.11 | .000 | .22 |  |
| Type | 1 | 99 | 12449.16 | 28235.55 | 43.65 | .000 | .31 |  |
| Group x Emo | 2 | 99 | 765.32 | 5766.18 | 6.57 | .002 | .12 |  |
| Group x Type | 2 | 99 | 7487.87 | 28235.55 | 13.13 | .000 | .21 |  |
| Emo x Type | 1 | 99 | 20.91 | 4811.24 | 0.43 | .513 | 0 |  |
| Group x Emo x Type | 2 | 99 | 583.02 | 4811.24 | 6.00 | .003 | .11 |  |

*Note.* Results of the repeated measures ANOVA for pleasantness ratings. *df_Num_* indicates degrees of freedom numerator. *df_Den_* indicates degrees of freedom denominator. *SS_Num_* indicates sum of squares numerator. *SS_Den_* indicates sum of squares denominator. *η^2^* indicates effect size.

| **sTable 2** | | | |
| --- | --- | --- | --- |
| *Post-Hoc Tests for Pleasantness Ratings (Image Type x Emotion x Group)* | | | |
| Emo vs. neut (three-way-interaction) | | | |
| Group | Type | p-value |  |
| HC | Objects | .1 |  |
| HC | Foods | .002* |  |
| AN | Objects | .07 |  |
| AN | Foods | .14 |  |
| BN | Objects | .25 |  |
| BN | Foods | .44 |  |
| Emo vs. neut (two-way-interaction) | | |  |
| HC |  | .01* |  |
| AN |  | .04* |  |
| BN |  | .97 |  |
| Foods vs. objects (two-way-interaction) | | |  |
| HC |  | <.001* |  |
| AN |  | .9 |  |
| BN |  | <.001* |  |
| *Note.* Post-Hoc tests for pleasantness ratings following up the Image Type X Emotion X Group interaction. * indicates significance at a threshold of *p <* .05. | | | |

| **sTable 3**  *Food cue reactivity (group x image type)* | | | |
| --- | --- | --- | --- |
| *Predictors* | *Estimates* | *std. Error* | *CI (95%)* |
| Intercept | -0.13 | 0.03 | -0.18 – -0.07 |
| Group AN | 0.08 | 0.04 | -0.00 – 0.15 |
| Group BN | 0.01 | 0.04 | -0.07 – 0.09 |
| Type | 0.04 | 0.04 | -0.03 – 0.11 |
| Type*Group AN | -0.22 | 0.05 | -0.33 – -0.11 |
| Type*Group BN | -0.09 | 0.06 | -0.20 – 0.02 |
| *Note.* Results of the Bayesian linear model for the two-way interaction Type x Group. | | | |
|  |  | | |

**sTable 4**

*Post-Hoc contrasts for the interaction image type x group of Cluster 1*

| Foods - Objects | | | |
| --- | --- | --- | --- |
| Group | estimate | HDI | p-direction |
| HC | -0.04 | -0.12 – 0.03 | 86.23 % |
| AN | 0.18 | 0.11 – 0.26 | 100 % |
| BN | 0.05 | -0-03 – 0.13 | 87.55 % |

*Note.* HDI: highest density interval; interval that contains the estimate with a probability of 95%. P-direction: probability that the effect lies in the direction of the estimate.

| **sTable 5** | | | |
| --- | --- | --- | --- |
| *Emotional food cue reactivity (group x image type x emotion)* | | | |
| Predictors | Estimates | std. Error | CI (95%) |
| Intercept | -0.06 | 0.06 | -0.18 – 0.05 |
| Group AN | 0.04 | 0.09 | -0.14 – 0.22 |
| Group BN | 0.08 | 0.09 | -0.10 – 0.25 |
| Type | 0.34 | 0.09 | 0.16 – 0.52 |
| Emo | 0.13 | 0.08 | -0.03 – 0.29 |
| Type*Group AN | -0.17 | 0.13 | -0.42 – 0.08 |
| Type*Group BN | -0.11 | 0.14 | -0.38 – 0.16 |
| Emo*Group AN | -0.13 | 0.12 | -0.37 – 0.11 |
| Emo*Group BN | -0.24 | 0.12 | -0.49 – -0.00 |
| Type*Emo | -0.33 | 0.12 | -0.57 – -0.08 |
| Type*Emo*Group AN | 0.51 | 0.18 | 0.16 – 0.86 |
| Type*Emo*Group BN | 0.31 | 0.19 | -0.05 – 0.68 |
| *Note.* Results of the Bayesian linear model for the  thre-way interaction Type x Group x Emo. | | | |

**sTable 6**

*Post-Hoc contrasts for the interaction image type x group x emotion of Cluster 3*

|  | Neutral – Negative | | | |
| --- | --- | --- | --- | --- |
| Group | Type | estimate | HDI | p-direction |
| HC | Objects | -0.13 | -0.29 – 0.03 | 94.73 % |
| HC | Foods | 0.2 | 0.01 – 0.38 | 98.05 % |
| AN | Objects | -0 | -0.18 – 0.18 | 50.53 % |
| AN | Foods | -0.19 | -0.37 - 0 | 97.3 % |
| BN | Objects | 0.12 | -0.07 – 0.29 | 88.48 % |
| BN | Foods | 0.13 | -0.07 – 0.33 | 90.48 % |

*Note.* HDI: highest density interval; interval that contains the estimate with a probability of 95%. P-direction: probability that the effect lies in the direction of the estimate.

**Additional exploratory analysis for high- and low-calorie foods in bulimia nervosa**

**sTable 7**

| *Food cue reactivity differentiating between high- and low-calorie foods in bulimia nervosa* | | | |
| --- | --- | --- | --- |
| *Predictors* | *Estimates* | *std. Error* | *CI (95%)* |
| Intercept | 0.01 | 0.02 | -0.02 – 0.04 |
| Low-Cal | -0.02 | 0.02 | -0.06 – 0.02 |
| Objects | -0.02 | 0.02 | -0.07 – 0.02 |
| *Note.* Results of the Bayesian linear model for image type (high-cal vs. low-cal vs. objects) in an early time window. Bulimia Nervosa (n = 32). | | | |
|  |  | | |

**sFigure 1**

*Food cue reactivity differentiating between high- and low-calorie foods in bulimia nervosa*

**
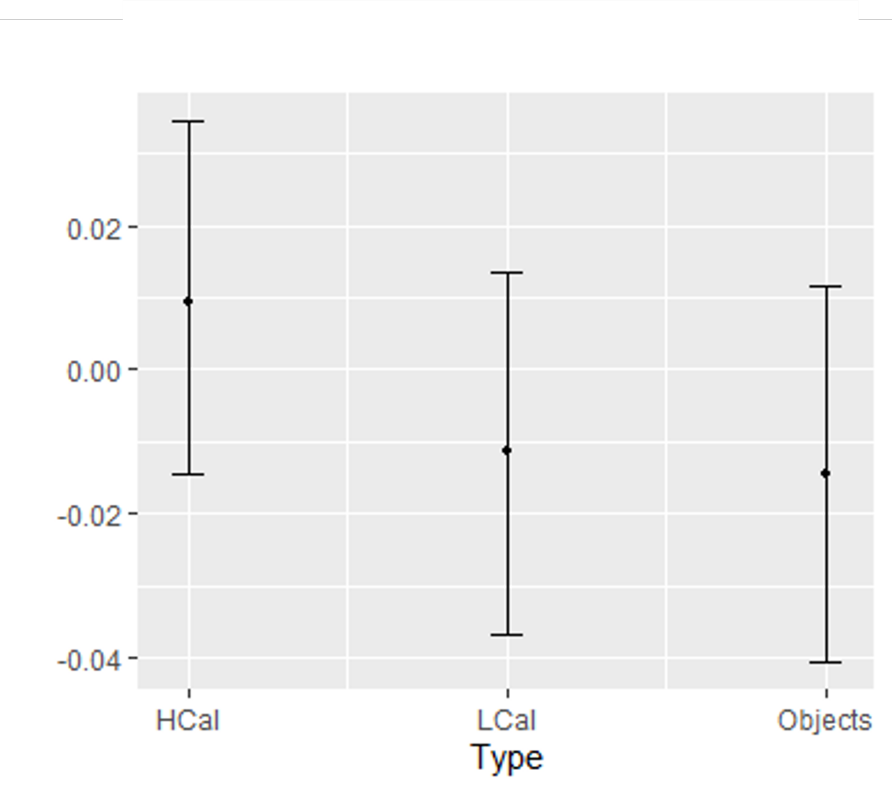
**

*Note.* Results of the linear model for the image type analysis in bulimia nervosa (n = 32). Beta values (y-axis) indicate the magnitude of influence of conditions on EEG amplitude.

| **sTable 8**  *Emotional food cue reactivity differentiating between high- and low-calorie foods in BN* | | | |
| --- | --- | --- | --- |
| *Predictors* | *Estimates* | *std. Error* | *CI (95%)* |
| Intercept | 0.02 | 0.02 | -0.02 – 0.06 |
| Low-Cal | -0.02 | 0.03 | -0.08 – 0.03 |
| Objects | -0.03 | 0.03 | -0.09 – 0.04 |
| Emo = Neutral | -0.02 | 0.03 | -0.08 – 0.04 |
| Low-Cal*Emo | 0 | 0.04 | -0.08 – 0.09 |
| Object*Emo | 0.01 | 0.04 | -0.08 – 0.1 |
| *Note.* Results of the Bayesian linear model for image type (high-cal vs. low-cal vs. objects) x emotions (negative vs. neutral) in BN. BN = Bulimia Nervosa (n = 32). | | | |
|  |  | | |

**sFigure 2**

*Emotional food cue reactivity differentiating between high- and low-calorie foods in BN*

**
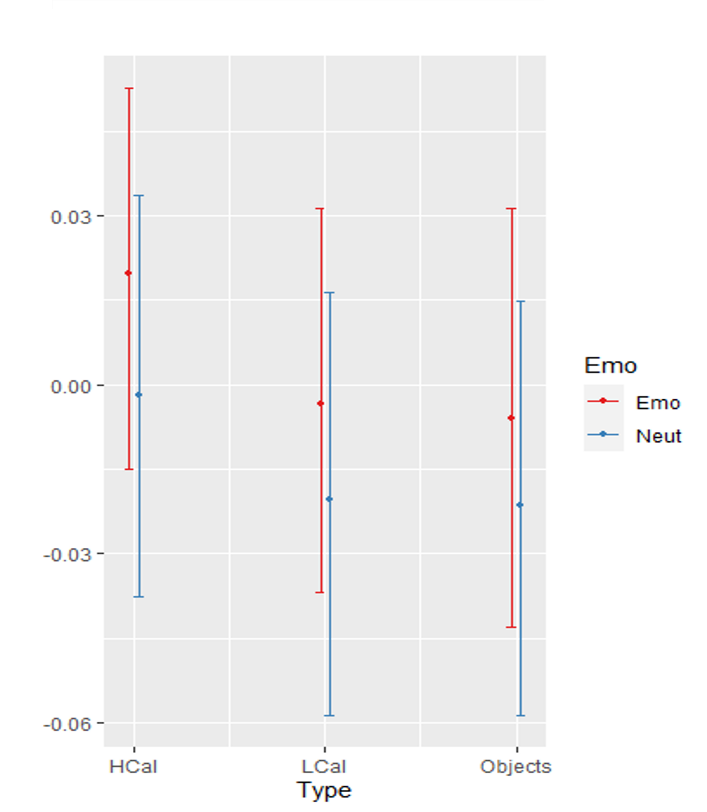
**

*Note.* Results of the linear model for the image type x emotion analysis in bulimia nervosa (n = 32). Beta values (y-axis) indicate the magnitude of influence of conditions on EEG amplitude.

Blechert, J., Lender, A., Polk, S., Busch, N. A., & Ohla, K. (2019). Food-Pics_Extended—An Image Database for Experimental Research on Eating and Appetite: Additional Images, Normative Ratings and an Updated Review. *Frontiers in Psychology*, *10*. https://doi.org/10.3389/fpsyg.2019.00307

Groppe, D. M., Urbach, T. P., & Kutas, M. (2011). Mass univariate analysis of event-related brain potentials/fields I: A critical tutorial review: Mass univariate analysis of ERPs/ERFs I: Review. *Psychophysiology*, *48*(12), 1711–1725. https://doi.org/10.1111/j.1469-8986.2011.01273.x

Luck, S. J., & Gaspelin, N. (2017). How to get statistically significant effects in any ERP experiment (and why you shouldn’t): How to get significant effects. *Psychophysiology*, *54*(1), 146–157. https://doi.org/10.1111/psyp.12639
